# Individual exposures to drinking water trihalomethanes, low birth weight and small for gestational age risk: a prospective Kaunas cohort study

**DOI:** 10.1186/1476-069X-10-32

**Published:** 2011-04-19

**Authors:** Regina Grazuleviciene, Mark J Nieuwenhuijsen, Jone Vencloviene, Maria Kostopoulou-Karadanelli, Stuart W Krasner, Asta Danileviciute, Gediminas Balcius, Violeta Kapustinskiene

**Affiliations:** 1 Department of Environmental Sciences, Vytautas Magnus University, Kaunas, Lithuania; 2 Center for Research in Environmental Epidemiology (CREAL), Barcelona, Spain; 3 Department of Marine Sciences, University of the Aegean, University Hill, Greece; 4 The Metropolitan Water District of Southern California, La Verne, CA, USA

## Abstract

**Background:**

Evidence for an association between exposure during pregnancy to trihalomethanes (THMs) in drinking water and impaired fetal growth is still inconsistent and inconclusive, in particular, for various exposure routes. We examined the relationship of individual exposures to THMs in drinking water on low birth weight (LBW), small for gestational age (SGA), and birth weight (BW) in singleton births.

**Methods:**

We conducted a cohort study of 4,161 pregnant women in Kaunas (Lithuania), using individual information on drinking water, ingestion, showering and bathing, and uptake factors of THMs in blood, to estimate an internal dose of THM. We used regression analysis to evaluate the relationship between internal THM dose and birth outcomes, adjusting for family status, education, smoking, alcohol consumption, body mass index, blood pressure, ethnic group, previous preterm, infant gender, and birth year.

**Results:**

The estimated internal dose of THMs ranged from 0.0025 to 2.40 mg/d. We found dose-response relationships for the entire pregnancy and trimester-specific THM and chloroform internal dose and risk for LBW and a reduction in BW. The adjusted odds ratio for third tertile vs. first tertile chloroform internal dose of entire pregnancy was 2.17, 95% CI 1.19-3.98 for LBW; the OR per every 0.1 μg/d increase in chloroform internal dose was 1.10, 95% CI 1.01-1.19. Chloroform internal dose was associated with a slightly increased risk of SGA (OR 1.19, 95% CI 0.87-1.63 and OR 1.22, 95% CI 0.89-1.68, respectively, for second and third tertile of third trimester); the risk increased by 4% per every 0.1 μg/d increase in chloroform internal dose (OR 1.04, 95% CI 1.00-1.09).

**Conclusions:**

THM internal dose in pregnancy varies substantially across individuals, and depends on both water THM levels and water use habits. Increased internal dose may affect fetal growth.

## Background

The association between exposure to disinfection by-products (DBPs), as measured by trihalomethanes (THMs), in drinking water and adverse reproductive/developmental effects has been extensively studied in recent epidemiological studies. Some epidemiological studies suggested that pregnant women exposed to water containing elevated THMs concentrations may be at greater risk for adverse pregnancy outcomes, including fetal growth, but findings of the studies to date have been inconsistent [1-3]. The relationship between DBP exposure and reproductive health outcomes remains unclear, mainly owing to limitations in the crude exposure assessment in most studies [4-8]. Epidemiological studies found mostly small increases in risk for low birth weight (LBW) at term or small for gestational age (SGA) [9-11] or yielded mixed results [12,13]. The epidemiological studies of reproductive outcomes have relied on different methods of assessing exposure, which presents difficulties in making comparisons between investigations and in generalizing results [6]. Recent studies have attempted to improve exposure assessment by using individual exposure measures combining routinely collected water system THM measurements with a measure of ingestion, such as number of glasses or water drank per day. However, only a few studies accounted for spatial and temporal fluctuations in THM levels across the distribution system over the time periods relevant to study pregnancy [14,15]. Furthermore, seeking to improve the exposure assessment, studies have begun to incorporate behavioral determinants of different routes of exposure to DBPs such as dermal absorption and inhalation during bathing and showering, and ingestion of drinking water but the contribution of these was unclear [16-18]. The recent epidemiological studies concluded that, while there appears to be suggestive evidence associating elevated total THM (TTHM) levels with some adverse reproductive outcomes, evidence for relationships with LBW and SGA are inconclusive and inconsistent, and further research is warranted, including on the importance of different exposure routes.

In the present study, we evaluated the effect of maternal THM dose on several indices of fetal development. Using prospective Kaunas cohort study with individual data, we were able to adjust for many important risk factors for LBW and SGA. Through improvements in individual THM exposure and dose assessment and controlling for many possible confounding variables, our study aims to offer estimated total individual internal dose assessment based on monitoring of tap water THM levels and detailed water use behaviors to examine dose-response relationships for THMs and fetal growth.

## Methods

### Participant recruitment and outcome assessment

We conducted a prospective cohort study of pregnant women in Kaunas city, Lithuania, as a part of the European Commission FP6 HiWATE Project Health impacts of long-term exposure to DBP in drinking water in Europe (HiWATE) [19].

On their first visit to a general practitioner, all pregnant women living in Kaunas city between 2007 and 2009 were invited to join the cohort. The women were enrolled in the study only if they consented to participate in the cohort. The study ethics complied with the Declaration of Helsinki [20]. The research protocol was approved by the Lithuanian Bioethics Committee and an oral informed consent was obtained from all subjects.

In total 5,405 women were approached; 79% of them agreed to participate in the study. The first interview was completed during the first pregnancy trimester. The median gestational age at interview was 8 weeks. The interview queried women regarding demographics, residence and job characteristics, chronic diseases, reproductive history, including date of last menstrual period, previous preterm delivery. We also asked the women to report their age (less than 20 years, 20-29 years, 30 years, and more), educational level (primary, secondary, university), marital status (married not married), smoking (non-smoker, smoker at least one cigarette per day), alcohol consumption (0 drinks per week, at least one drink per week), blood pressure (<140/80 mm/Hg, ≥140 or ≥ 90 mm/Hg), body mass index (<25 kg/m^2^, 25-30 kg/m^2^, >30 kg/m^2^), and other potential risk factors for LBW. Adjustment for these variables was made for studies of various birth outcomes subgroups. The women also were examined by ultrasound to determine the gestational age of the fetus.

A special water consumption and water use habits questionnaire was used to interview the 4,260 women who agreed to participate in the study; 76.4% of them were interviewed during the third pregnancy trimester before delivery at the hospital and 23.6% by telephone within the first month after delivery. Consumption was ascertained for three types of water: cold tape water or dinks made from cold tap water; boiled tap water (tea, coffee, and other); and bottled water, used at home, at work, other. In addition, number of showers, baths, swimming pools weekly, and their average length was asked of all subjects. The interviews were conducted by trained nurses who did not know the THM exposure status and birth outcome.

Pregnancy outcomes were abstracted from the medical records. LBW were defined as infant's BW less than 2,500 g. Infants were considered SGA if they were in the lowest 10th centile of BW for each gestational week stratified by infant gender and maternal ethnic group. Gender-specific and ethnic group-specific deciles were determined from the 2004 data set of all births in Lithuania [21]. Women with multiple pregnancies (150), having inconsistent or invalid data for dating the pregnancy (5) or estimating THM exposure (mostly students moved out of the city during pregnancy, 839) or with newborn BW above 4,500 g (75) were excluded. We restricted our analyses to infants born with a BW below 4,500 g, leaving data for 3,341 women in the final analysis.

We also conducted analyses comparing questionnaire data and birth certificate data on various characteristics among participants and non-participants. The mean BW, gestational duration, prevalence of LBW and SGA were similar among the two groups. These two groups did not differed by ethnic group, consumption tap water, showering, and bathing, however, nonparticipating mothers were younger (<20 years, 3.9% vs. 1.8%), less educated (did not graduate from university, 46.6% vs. 54.3%), more often smokers (smokers, 9.6% vs. 6.9%), and did have fewer prior births (no child, 64.1% vs. 45.1%), than that of participants. In addition, to assess the level of accuracy in personal reporting that can bias the THM risk estimates, questionnaire information was collected repeatedly on 10% subjects. There were no significant differences in reporting water use habits and other covariates.

### Exposure Assessment

The Kaunas city municipal drinking water is supplied by four water treatment plants system. The each treatment plant water supplied system is constituted of only one sub-system (i.e., one chlorination, and branchy water supplied to the users). Groundwater sources are used for the whole water supply system.

However, the four water treatment plants, which disinfected ground water with sodium hypochlorite (chlorine dose 0.26-0.91 mg/L, residual chlorine 0-0.22 mg/L), produced different concentrations of THMs in finished water. One treatment plant (Petrasiunai) supplied finished water with higher levels of THMs ("high level THM site," 54.9% subjects), and the three other plants supplied finished water with lower levels of all THMs ("low level THM site"). Water samples were collected four times per year over a 3-year study period (2007-2009) in the morning in three locations: close to the treatment plant, at 5 km, and at 10 km or more from every treatment plant. A total of 85 water samples were collected from 12 monitoring sites in four water supply zones for THM analysis. Samples were analysed at the University of the Aegean, Greece, by using gas chromatography with electron capture detection [22]. Measurements included specific values for the four regulated THMs (chloroform, bromoform, bromodichloromethane, and dibromochloromethane) and nine haloacetic acids (HAAs). Selected samples were analyzed for five haloacetonitriles, two haloketones, chloropicrin, and chloral hydrate. In addition, selected samples were analyzed at the National Institute for Health and Welfare (THL), Finland, for the halogenated furanone MX. Only THMs data were evaluated in this study since the other halogenated DBPs were present only at low or sub μg/L levels, if detected at all.

We calculated the mean quarterly THM constituent concentrations for water zones and subsequently, depending on the TTHM levels within each zone, assigned "low level" and "high level" sites. We used tap water THM concentration, derived as the average of quarterly sample values over the time that the pregnancy occurred from all sampling sites located in the each distribution system, and geocoded maternal address at birth to assign the individual women residential exposure index. Estimates of exposure index to total and specific THMs from drinking water were tabulated first as an average level at the tap over the pregnancy period; this measure was then categorized at the tertiles of the distribution for birth outcomes. In addition, trimester-specific analyses were conducted.

We combined every subject's residential exposure index and water-use questionnaire data to assess individual exposure through ingestion of THMs. Women were asked to indicate the cup or glass size and number of cups or glasses of tap water consumed per day, including hot and cold beverages made from tap water. With this information, we calculated daily amounts of hot and cold tap water ingested. Integration of the information on residential THM levels (μg/L), ingested amounts (L/day), and modifications by heating using an estimated uptake factor of 0.00490 to derive an integrated index of blood concentration, expressed in micrograms per day (mg/d) [18,23].

The actual algorithms of internal dose from ingestion were chloroform level (μg/l) × water consumption (l/day) × 0.00490196 μg/μg/l; brominated THM level (μg/l) × water consumption (l/day) × 0.00111848 μg/μg/l.

We assumed a null THM level for any bottled water consumption since in local bottled water production chlorination and ozonation is not used.

Finally, we addressed dermal absorption and inhalation by considering showering and bathing alone and combined with ingestion. We multiplied residential THM levels (μg/L) by frequency and average duration of bathing or showering per day (min/day) and calculated each mother's trimester-specific and entire pregnancy average daily uptake of THM internal dose (mg/d). We derived indices of daily uptake by integrating THM concentrations, duration of bathing and showering reported in a questionnaire administered to study participants, and estimated uptake factors of 0.001536 and 0.001321 of THMs in blood per minute per microgram from showering and bathing, respectively [24,25]. The uptake factors of THMs individual constituents were assessed on the relative changes in blood levels after 10 minutes exposure (after versus before ingestion 1 L of tap water, 10 minutes showering, and 10 minutes bathing).

The actual algorithms of internal dose from showering and bathing were min/day showering × μg/l chloroform in water × 0.001536261 μg/min/μg/l, min/day showering × μg/l brominated THM in water × 0.001352065 μg/min/μg/l, min/day bathing × μg/l chloroform in water × 0.001320755 μg/min/μg/l, min/day bathing × μg/l brominated THM in water × 0.00129571 μg/min/μg/l

We then used average daily total uptakes in our analysis as continuous and categorized variables. We calculated tertiles of THM internal dose. This gave first (0.0025-0.0386 mg/d), second (0.0386-0.3496 mg/d), and third (0.3496-2.4040 mg/d) tertiles for average TTHM uptake. To reduce exposure misclassification errors in the subsequent analysis, we used a subset of women who through the entire pregnancy did not change their address.

### Analysis

The data analysis compared the LBW, SGA, and BW of low, medium and high exposed women. We used logistic regression to estimate adjusted odds ratios (ORs) and 95-percent confidence intervals (CIs) for LBW, SGA, and the various exposure indices. We categorized TTHM internal dose in tertiles and evaluated the possible relationship between increases in adverse birth outcomes risk for an increase in estimated TTHM internal dose. We ran multivariate logistic regression models for the TTHMs, chloroform, dibromochloromethane, and bromodichloromethane for the total pregnancy and trimester-specific periods. We also used multiple linear regressions for TTHM internal dose analysis as continuous variable to evaluate the relationship, if any between BW reductions and every 1 μg/d increase in TTHM internal dose.

Risk factors for LBW have been reported extensively elsewhere [26,27] and are not the subject of this article, except to allow for appropriate control of covariates in this analysis. In the logistic regression models for adverse birth outcomes, using personal data of the cohort sample, we assessed a variety of potential confounders identified by univariate analysis. Further, we examined the association of THM exposure and birth outcomes with a multivariable analysis controlling for effect of major covariates that changed the adjusted ORs for THM by 10% or more. The adjusted birth outcomes analyses included family status, maternal education, chronic diseases, body mass index, blood pressure, smoking, alcohol consumption, ethnicity, previous preterm delivery, infant gender, and birth year. Two-tailed statistical significance was evaluated by using a p value of 0.05. All statistical analyses were carried out using the SPSS software for Windows version 12.0.1.

## Results

The mean TTHM level in the low level site from three water treatment plants was 1.3 μg/L, and in the high level site (Petrasiunai) 21.9 μg/L (Table 1). The yearly and seasonal fluctuations in the levels of TTHMs were primarily the result of the lack of THM formation for Petrasiunai in March, 2008. There was little spatial and temporal variability within the high and low areas.

**Table 1:** THM levels (μg/L) by sampling site, water supply zone, year and season of sampling

Tap water sampling	**TTHMs**^**c**^**Mean (SD**^**d**^**)**	**CHCl**_**3**_**Mean (SD)**	**CHBr**_**2**_**Cl Mean (SD)**	**CHBrCl**_2 _**Mean (SD)**
Sampling sites				

All sites	9.8 (12.4)	7.8 (10.2)	0.3 (0.5)	1.7 (2.2)
Low THM level^a^	1.3 (1.2)	0.9 (1.0)	0.1 (0.2)	0.3 (0.5)
High THM level^b^	21.9 (10.9)	17.7 (9.0)	0.5 (0.6)	3.6 (2.1)

Year of sampling				

2007 all sites	10.3 (13.5)	8.7 (12.0)	0 (0 ^e^)	1.5 (1.6)
Low THM level^a^	0.9 (1.3)	0.39 (1.0)	0 (0)	0.6 (0.5)
High THM level^b^	24.2 (11.0)	21.3 (9.6)	0 (0)	2.9 (1.7)

2008 all sites	6.2 (10.2)	4.4 (7.5)	0.3 (0.5)	1.5 (2.4)
Low THM level^a^	1.5 (1.1)	0.9 (0.6)	0.2 (0.3)	0.5 (0.5)
High THM level^b^	12.7 (13.5)	9.3 (9.8)	0.6 (0.6)	2.8 (3.3)

2009 all sites	11.8 (12.8)	9.5 (10.0)	0.4 (0.5)	1.9 (2.3)
Low THM level^a^	1.3 (1.1)	1.3 (1.0)	0.1 (0.2)	0.1 (0.2)
High THM level^b^	26.5 (2.9)	21.0 (2.3)	0.9 (0.4)	4.6 (0.5)

Season of sampling				

Spring all sites	8.5 (12.1)	6.8 (9.7)	0.3 (0.4)	1.4 (2.1)
Low THM level^a^	1.4 (1.3)	1.2 (1.1)	0.1 (0.3)	0.2 (0.4)
High THM level^b^	18.3 (13.6)	14.7 (10.9)	0.5 (0.5)	3.1 (2.3)

Summer all sites	9.9 (12.7)	8.3 (11.3)	0 (0)	1.6 (1.7)
Low THM level^a^	1.0 (1.4)	0.4 (1.0)	0 (0)	0.7 (0.5)
High THM level^b^	24.1 (8.3)	21.0 (7.0)	0 (0)	3.1 (2.0)

Autumn all sites	11.1 (13.4)	8.8 (11.1)	0.2 (0.5)	2.0 (2.4)
Low THM level^a^	1.2 (1.1)	0.8 (0.9)	0 (0)	0.4 (0.5)
High THM level^b^	24.8 (9.7)	20.1 (8.6)	0.6 (0.6)	4.2 (2.4)

Winter all sites	10.9 (12.1)	8.4 (9.3)	0.5 (0.6)	1.9 (9.3)
Low THM level^a^	1.1 (1.0)	0.9 (0.6)	0.1 (0.3)	0.1 (0.3)
High THM level^b^	24.5 (1.4)	18.9 (1.2)	1.1 (0.1)	4.5 (0.2)

^a^Viciunai, Eiguliai, Kleboniskis. ^b^Petrasiunai.

^c^TTHMs = total trihalomethanes: the sum of CHCl_3 _(chloroform),

CHBr_2_Cl (dibromochloromethane), and CHBrCl_2 _(bromodichloromethane).

^d^SD = standard deviation. ^e^0 = below the limit of detection.

Chloroform was the dominant THM species in this water, contributing approximately 80% of the mass of the TTHMs. The brominated THM species were significantly lower: dibromochloromethane ranged from 0.06 to 0.5 μg/L and bromodichloromethane ranged from 0.3 to 3.6 μg/L. Bromoform was below the limit of detection. The correlation between individual THM concentrations was high (r = 0.91-0.99, p = 0.000) and the correlations between each pregnancy trimester ranged from 0.62 to 0.96, (p = 0.000).

Although there was a difference in the concentrations of TTHMs between Petrasiunai and that of the other sites, there was no difference in the levels of the other halogenated DBPs, which were present at low or sub μg/L levels, if detected at all. The mean sum (and standard deviation) of the dihalogenated and trihalogenated HAAs for Petrasiunai was 0.5 (0.7) and 0.3 (0.7) μg/L, respectively; whereas they were 0.3 (0.8) and 0.1 (0.2) μg/L, respectively, for the other sites. The mean values of other individual halogenated DBPs (i.e., haloacetonitriles, haloketones, chloropicrin, chloral hydrate, monohalogenated HAAs) were all less than 1.0 μg/L each for Petrasiunai and the other sites. MX was only measured once for Petrasiunai and it was not detected, whereas it was measured three times in the other sites and was 0.6-1.5 ng/L. Thus, only THM data were evaluated in this analysis, since there was a substantial difference in THM occurrence between Petrasiunai and the other sites.

The women recruited were predominantly Lithuanian in ethnic origin (97.4%) and did not smoke (93.1%). The mean age at enrolment was 28.4 years, and the women tended to be highly educated (54.3% with a university degree). The mean BW of the 3,341 singleton infants included in our analysis was 3,445 g. Among these, 156 (4.7%) were classified as LBW and 270 (8.1%) as SGA. The vast majority of SGA infants (93.0%) were term births (37 weeks or above). In general, mothers who smoked, were single, less educated, had previous preterm delivery, or suffered from a disease during pregnancy delivered a higher proportion of LBW or SGA infants. We did not find a difference in fetal growth between water filter users and non-users. The analysis by TTHM internal dose tertiles showed, that most characteristics of the exposure groups were similar (Table 2). There were no differences in social and demographic characteristics, health behaviour, pregnancy history, and maternal diseases. However, paternal smoking and alcohol consumption differed between exposure groups. The proportion of LBW cases increased with increasing THM exposure (3.7, 4.4 and 5.9%, respectively, low, medium and high exposure). We also found an increase in the proportion of SGA cases (7.0, 8.0 and 9.2%, respectively).

**Table 2:** Distribution of Kaunas cohort study subjects for various characteristic by exposure

Risk factor/outcome	Low THM N (%)	Medium THM N (%)	High THM N (%)
Maternal age			
< 20 years	19 (1.8)	17 (1.5)	23 (2.1)
20-29 years	652 (60.1)	688 (59.7)	658 (59.6)
≥ 30 years	414 (39.2)	447 (38.8)	423 (38.3)

Marital status			
Married	876 (80.7)	958 (83.2)	910 (82.4)
Not married	209 (19.3)	194 (16.8)	194 (17.6)

Maternal education			
Primary school	59 (5.4)	50 (4.3)	57 (5.2)
Secondary school	454 (41.8)	465 (40.4)	442 (40.0)
University degree	572 (52.7)	637 (55.3)	605 (54.8)

Maternal smoking			
Nonsmoker	1003 (92.4)	1076 (93.4)	1031 (93.4)
Smoker	82 (7.6)	76 (6.6)	73 (6.6)

Paternal smoking^a^			
Nonsmoker	574 (53.4)	629 (55.4)	545 (49.8)
Smoker	501 (46.6)	507 (44.6)	550 (50.2)

Alcohol consumption^a^			
No	1000 (92.2)	1094 (95.0)	1048 (94.9)
Yes	85 (7.8)	58 (5.0)	56 (5.1)

Blood pressure			
<140/80 mm/Hg	969 (89.3)	1020 (88.5)	977 (88.5)
≥140 or ≥ 90 mm/Hg	116 (10.7)	132 (11.5)	127 (11.5)

Ethnic group			
Lithuanian	1054 (97.1)	1117 (97.0)	1082 (98.1)
Other	31 (2.9)	35 (3.0)	21 (1.9)

Parity			
No child	492 (45.3)	499 (43.3)	516 (46.7)
≥ 1 child	593 (54.7)	653 (56.7)	588 (53.3)

Infant gender			
Male	559 (51.5)	611 (53.0)	544 (49.3)
Female	526 (48.5)	541 (47.0)	560 (50.7)

Current residence			
1-4 years	437 (40.3)	492 (42.7)	472 (42.8)
5-9 years	257 (23.7)	288 (25.0)	296 (26.8)
≥ 10 years	391 (36.0)	372(32.3)	336 (30.4)

Work exposure			
No	996 (91.8)	1053 (91.4)	999 (90.5)
Yes	89 (8.2)	99 (8.6)	105 (9.5)

Chronic disease			
No	825 (76.0)	858 (74.5)	844 (76.4)
Yes	260 (24.0)	294 (25.5)	260 (23.6)

Previous preterm delivery			
No	1069 (98.5)	1123 (97.5)	1087 (98.5)
Yes	16 (1.5)	29 (2.5)	17 (1.5)

Socio economic status			
Low income	335 (30.9)	337 (29.3)	338 (30.6)
Medium income	582 (53.6)	642 (55.7)	600 (54.3)
High income	168 (15.5)	173 (15.0)	166 (15.0)

Body mass index (kg/m^2^)			
<25 Normal	618 (57.0)	677 (58.8)	679 (61.5)
25-30 Overweight	329 (30.3)	334 (29.0)	284 (25.7)
30 Obesity	138 (12.7)	141 (12.2)	141 (12.8)

Water filter			
Yes	341 (31.4)	336 (29.2)	338 (30.6)
No	744 (68.6)	816 (70.8)	766 (69.4)

LBW^a^			
Yes	40 (3.7)	51 (4.4)	65 (5.9)
No	1045 (96.2)	1101 (95.7)	1039 (94.1)

SGA			
Yes	76 (7.0)	92 (8.0)	102 (9.2)
No	1009 (93.0)	1060 (92.0)	1002 (90.8)

Mean birth weight (SDs)	3449 (517)	3462 (524)	3430 (559)

^a^p < 0.05.

Municipal water was the drinking water source of all study subjects. Fifty-two percent of the women consumed tap water and 12% of women reported consumption of other tap-water beverages. Overall, women consumed an average of 0.79 L of cold tap water, 1.04 L of boiled water, and 1.09 L of bottled water per day. The cohort study subjects' daily water intake for water users of low, medium, and high THM exposure was similar (Table 3). The highest amount of tap water was consumed at home (0.62, 0.65, and 0.69 L, respectively, low, medium, and high exposure), while at work and in other places, tap water usage was low (mean 0.1 L).

**Table 3:** Summary of Kaunas cohort study subjects daily water intake for water users by THM exposure

Mean daily ingestion (L/day)	Low THM		Medium THM		High THM	
	**Mean**	**SD**	**Mean**	**SD**	**Mean**	**SD**

Consumption tap water (52.1%)^a^						
At home	0.62	0.43	0.65	0.48	0.69	0.49
At work	0.10	0.23	0.10	0.24	0.11	0.25
Other	0.02	0.09	0.02	0.09	0.02	0.11
In total	0.74	0.52	0.77	0.11	0.82	0.60

Other tap-water beverages (12.2%)^a^						
At home	0.36	0.35	0.39	0.37	0.37	0.38
At work	0.09	0.18	0.07	0.17	0.07	0.15
Other	0.05	0.12	0.07	0.21	0.06	0.16
In total	0.50	0.37	0.53	0.42	0.50	0.41

Consumption bottled water (78.1%)^a^						
At home	0.61	0.51	0.70	0.54	0.61	0.56
At work	0.35	0.38	0.38	0.40	0.39	0.39
Other	0.06	0.16	0.06	0.20	0.06	0.17
In total	1.01	0.69	1.14	0.75	1.07	0.76

Boiled water (tea, coffee) (95%)^a^						
At home	0.51	0.30	0.53	0.30	0.51	0.32
At work	0.28	0.29	0.29	0.29	0.30	0.28
Other	0.05	0.13	0.05	0.14	0.05	0.13
In total	0.84	0.47	0.87	0.46	0.87	0.46

^a^% of individuals reporting daily water ingestion.

Showering was common (96% of subjects) and 37% took either shower or a bath during the pregnancy. Mean frequency of showering was 6.5 times per week, with a mean duration of 15.2 minutes per shower. Average frequency of bathing was 1.8 times per week, with a mean duration of 33.5 minutes per bath. The percentage of participants who attended swimming pools was low (7%). The reporting of showering and bathing increased with increasing THM exposure. The mean time of showering was 69.73 minutes per week in the low-exposure group, 92.21 minutes per week in the medium exposure group, and 114.33 minutes per week in the high exposure group. Mean bathing time also increased with increasing exposure from 42.64 minutes per week to 63.53 minutes per week.

THM integrated uptake included ingestion, showering, and bathing. Uptake via ingestion contributed 8%; showering and bathing were the main contributors for TTHM and made up 92% of the total internal dose. The variability in frequency and duration of showering and bathing determined the TTHM internal dose variability.

The individual total uptake of TTHMs ranged between 0.0025 and 2.40 mg/d. The total chloroform uptake ranged between 0.0013 and 2.13 mg/d. Mothers supplied with water who had a higher chloroform concentration generally also had a higher total internal dose, and mothers supplied with water that had a lower chloroform concentration generally also had a low total internal dose. Daily uptake of bromodichloromethane ranged between 0.0001 and 0.34 mg/d and dibromochloromethane ranged between 0 and 0.064 mg/d.

We found a correlation between total pregnancy daily uptake tertile dose levels of TTHMs and trimester-specific levels. The correlation coefficient between TTHM uptake in the first and second trimester was 0.98, p < 0.001, and between the first and third trimester was 0.95, p < 0.001. A similar strong correlation was found for the uptake of THM constituents between the pregnancy trimesters (r = 0.99-0.81). The strong correlation is a result of limited variability in the amount of THMs produced from season to season at these groundwater treatment plants.

Exposure to TTHMs was associated with an increased risk for LBW using tertiles and a reduction in BW using a continuous variable (Table 4). After adjustment for potential confounding factors, we observed a statistically significant increased risk with higher dose levels (second and third tertiles) of TTHMs during the three trimesters and entire pregnancy. During the entire pregnancy, the odds ratios for LBW were 1.77, 95% CI 0.95-3.30; and OR 2.13, 95% CI 1.17-3.87, respectively, for second and third tertiles compared to the first tertile. The LBW risk (OR) observed per 0.1 μg/d increase in TTHMs was 1.08, 95% CI 1.01-1.16 and 1.07, 95% CI 1.00-1.15; and decrease in BW was 49.3 g (-146.3 to -1.5) and 47.2 g (-92.7 to -1.6) during the entire pregnancy and third trimester. Similarly, first, second, and third trimesters chloroform dose categories were associated with a statistically significant increase in the risk for LBW. Chloroform analyzed as continuous variable (increase of 0.1 μg/d) was also associated with an increase in risk for LBW (OR 1.10, 95% CI 1.01-1.19) for the entire pregnancy, as well as for trimester-specific time windows. In a linear regression we found a mean decrease in BW of 59.3 g (-114.8 to -3.7) for the entire pregnancy and 57.8 g (-111.6 to -4.0) for the third trimester, respectively, with increasing dose levels of chloroform. For bromodichloromethane, we observed statistically significant increases in LBW risk for the third tertile compared to the first tertile for the third trimester, OR 1.80, 95% CI 1.00-3.26. For bromodichloromethane internal dose as a continuous variable, we found an elevated risk in LBW for the entire pregnancy, first and third trimesters (ORs 1.04-1.05 for an increase of every 0.01 μg/d). The dibromochloromethane internal dose results were statistically significant for entire pregnancy and third trimester (OR 2.52, 95% CI 1.00-6.36 and OR 2.42, 95% CI 1.03-5.66, respectively). No significant reduction in BW as a continuous variable was found.

**Table 4:** LBW adjusted odds ratio and BW change for gestational exposure to internal dose THMs

THM exposure Tertile limits (mg/d)	LBW cases	Non LBW	**Entire pregnancy OR**^**a **^**(95% CI)**	First trimester OR (95% CI)	Second trimester OR (95% CI)	Third trimester OR (95% CI)
TTHM ^b^						
0.0025-0.0386	40	1046	Reference	Reference	Reference	Reference
0.0386-0.3545	51	1099	1.77 (0.95-3.30)	1.94 (1.04-3.62)	1.71 (0.92-3.18)	2.31 (1.22-4.35)
0.3545-2.4040	65	1040	2.13 (1.17-3.87)	2.29 (1.24-4.22)	2.06 (1.14-3.73)	2.12 (1.14-3.92)
Continuous (0.1 μg/d)			1.08 (1.01-1.16)	1.08 (1.01-1.16)	1.07 (1.00-1.15)	1.07 (1.00-1.15)
Change in BW^c ^g			-49.3^d ^(-146.3 - -1.5)	-45.7^d ^(-91.4-0.0)	-45.3 (-92.8-2.2)	-47.2^d ^(-92.7 - -1.6)

Chloroform						
0.0013-0.0249	40	1050	Reference	Reference	Reference	Reference
0.0249-0.2868	52	1093	2.06 (1.10-3.85)	2.30 (1.21-4.36)	1.79 (0.95-3.36)	2.12 (1.11-4.02)
0.2868-2.1328	64	1042	2.17 (1.19-3.98)	2.41 (1.30-4.49)	2.13 (1.18-3.85)	2.13 (1.15-3.92)
Continuous (0.1 μg/d)			1.10 (1.01-1.19)	1.10 (1.02-1.18)	1.10 (1.01-1.18)	1.09 (1.01-1.18)
Change in BW^c ^g			-59.3^d ^(-114.8 - -3.7)	-52.8^d ^(-104.4 - -1.2)	-53.4 (-108.2-1.3)	-57.8^d ^(-111.6 - -4.0)

CHBrCl_2_						
0.0001-0.0124	45	1046	Reference	Reference	Reference	Reference
0.0124-0.0501	53	1093	1.83 (1.01-3.34)	1.93 (1.05-3.55)	1.95 (1.07-3.58)	1.64 (0.89-3.02)
0.0501-0.3359	58	1046	1.64 (0.90-2.98)	2.06 (1.11-3.80)	1.82 (0.99-3.35)	1.80 (1.00-3.26)
Continuous (0.01 μg/d)			1.05 (1.00-1.11)	1.05 (1.00-1.11)	1.05 (0.99-1.11)	1.04 (1.00-1.10)
Change in BW^e ^g			-28.2 (-63.2-6.9)	-29.7 (-67.5-8.0)	-27.7 (-63.2-7.7)	-25.7 (-57.2-5.8)

CHBr_2_Cl						
0.0000-0.0000	57	1058	Reference	Reference	Reference	Reference
0.0000-0.0039	49	1075	3.02 (1.23-7.40)	0.62 (0.28-1.37)	0.68 (0.31-1.46)	2.44 (1.05-5.70)
0.0039-0.0644	50	1052	2.52 (1.00-6.36)	0.74 (0.37-1.49)	0.78 (0.36-1.67)	2.42 (1.03-5.66)
Continuous (0.01 μg/d)			1.18 (0.85-1.65)	1.06 (0.73-1.54)	1.16 (0.84-1.61)	1.23 (0.93-1.61)
Change in BW^e ^g			-24.3 (-215.7-167.2)	15.5 (-197.0-228.1)	-18.8 (-203.3-165.7)	-45.9 (-207.6-114.8)

^a^adjusted for squared gestational age, marital status, maternal education, chronic diseases, body mass index, blood pressure, smoking, alcohol consumption, previous preterm delivery, infant gender, and birth year.

^b^TTHM, total trihalomethane; CHBrCl_2_, bromodichloromethane; CHBr_2_Cl, dibromochloromethane.

^c^Change in birth weight in grams, of infants below 3,500 g, for every 1 μg/d increase in THMs internal dose.

^d^p < 0.05.

^e^Change in birth weight in grams, of infants below 3,500 g, for every 0.1 μg/d increase in THMs internal dose.

We found slight a increase in the risk of SGA related to elevated internal doses of THMs, hovewer, the results were statistically non-significant (Table 5). We observed slight increases in the risk for SGA among TTHMs exposed women (ORs 1.13-1.34). Chloroform dose was also associated with a slight increases in the risk of SGA (OR 1.19, 95% CI 0.87-1.63 and OR 1.22, 95% CI 0.89-1.68, respectively, for second and third tertile of third trimester and OR 1.04, 95% CI 1.00-1.09 per every 0.1 μg/d increase in the chloroform internal dose). Bromodichloromethane internal dose was associated with an increased risk but this was not monotonic (OR 1.37, 95% CI 1.00-1.88 and OR 1.25, 95% CI 0.91-1.73, respectively, for second and third tertile of third trimester), and it was not statistically significant as a continuous variable (OR 1.20, 95% CI 0.90-1.62).

**Table 5:** SGA adjusted odds ratio for gestational exposure to internal dose THMs

THM exposure Tertile limits (mg/d)	SGA cases	Non SGA	**Entire pregnancy OR**^**a **^**95% CI**	**First trimester OR**^**a **^**95% CI**	**Second trimester OR**^**a **^**95% CI**	**Third trimester OR**^**a **^**95% CI**
TTHM^b^						
0.0025-0.0386	76	1010	Reference	Reference	Reference	Reference
0.0386-0.3545	92	1058	1.18 (0.86-1.82)	1.13 (0.82-1.54)	1.18 (0.86-1.62)	1.17 (0.85-1.60)
0.3545-2.4040	102	1003	1.34 (0.98-1.84)	1.27 (0.93-1.73)	1.33 (0.97-1.82)	1.22 (0.89-1.67)
Continuous 0.1 μg/L			1.03 (0.99-1.07)	1.02 (0.98-1.06)	1.03 (0.99-1.07)	1.02 (0.98-1.06)

Chloroform						
0.0013-0.0249	78	1012	Reference	Reference	Reference	Reference
0.0249-0.2868	91	1054	1.16 (0.84-1.59)	1.19 (0.86-1.63)	1.12 (0.82-1.54)	1.19 (0.87-1.63)
0.2868-2.1328	101	1005	1.31 (0.96-1.79)	1.30 (0.95-1.77)	1.28 (0.94-1.74)	1.22 (0.89-1.68)
Continuous 0.1 μg/L			1.03 (0.99-1.08)	1.03 (0.99-1.08)	1.03 (0.99-1.08)	1.04 (1.00-1.09)

CHBrCl_2_						
0.0001-0.0124	73	1018	Reference	Reference	Reference	Reference
0.0124-0.0501	101	1045	1.35 (0.99-1.86)	1.21 (0.88-1.67)	1.20 (0.88-1.65)	1.37 (1.00-1.88)
0.0501-0.3359	96	1008	1.29 (0.94-1.78)	1.35 (0.99-1.85)	1.23 (0.90-1.69)	1.25 (0.91-1.73)
Continuous 0.01 μg/L			1.21 (0.90-1.62)	1.19 (0.87-1.62)	1.19 (0.89-1.60)	1.20 (0.90-1.62)

CHBr_2_Cl						
0.0000-0.0000	102	1013	Reference	Reference	Reference	Reference
0.0000-0.0039	78	1045	0.77 (0.56-1.05)	0.76 (0.54-1.07)	0.66 (0.48-0.91)	1.76 (0.56-1.03)
0.0039-0.0644	90	1012	0.89 (0.66-1.21)	0.88 (0.67-1.17)	0.90 (0.68-1.21)	0.85 (0.63-1.15)
Continuous 0.01 μg/L			1.05 (0.89-1.24)	1.05 (0.86-1.26)	1.05 (0.86-1.26)	1.06 (0.92-1.22)

^a^adjusted for: previous preterm delivery, maternal education, marital status, smoking, alcohol consumption, body mass index, maternal age, parity, and birth year.

^b^TTHM, total trihalomethane; OR, odds ratio; CI, confidence intervals; CHBr_2_Cl, dibromochloromethane; CHBrCl_2_, bromodichloromethane.

## Discussion

We conducted a prospective cohort study to examine the effects of internal dose of THM during the entire pregnancy and during three trimesters on LBW, BW, and SGA births. We observed a low spatial variation in THM levels measured in three locations: close to the treatment plant, at 5 km and at 10 km or more from every treatment plant in the each distribution system. The low spatial variability of TTHM present in Kaunas groundwater distribution systems could be explained by the relatively simple structure (i.e. one subsystem) and low presence of DBPs precursors at the groundwater sources [4,28]. Personal behavior was the main determinant of exposure variability of the study subjects. Uptake via showering and bathing provided a greater contribution to the uptake of the TTHM to the internal dose than did ingestion of tap water (92 and 8%, respectively). We demonstrated consistent, statistically significant effects of THM exposure on LBW and BW with an indication of dose-response relation. We found both excess risk of LBW during the entire pregnancy and during three trimesters as well. Specifically, there was a statistically significant excess risk of LBW for those exposed to higher internal doses of TTHM and chloroform in the three trimesters and a slight excess risk for those exposed to higher internal doses of bromodichloromethane and dibromochloromethane during the entire pregnancy and during third trimester. TTHM constituents (chloroform, bromodichloromethane and dibromochloromethane) analysed as categorical variables showed a slight excess risk of SGA during the entire pregnancy as well as trimester-specific periods. The probability of delivering an SGA infant was elevated by 4% per every 0.1 μg/d increase in the chloroform internal dose during the third trimester pregnancy. The lack of statistically significant effects for other TTHM constituents may be due to low exposure because of low levels, and lack of power in our study sample.

Although the third trimester is the most important in terms of fetal body mass growth, it has been hypothesized that early-pregnancy exposure may hamper fetal growth [15]. Therefore we conducted analyses exploring effects for three trimester-specific gestational exposures and entire pregnancy exposures. In our analyses it was more difficult to evaluate any independent effects of the trimesters because of the high correlation in exposure between them.

The epidemiological evidence for an association between exposure to THM and indicators of fetal growth is relatively inconsistent. A number of prior investigations have evaluated crude exposure during the third trimester of pregnancy, the time period of gestation when fetal growth may be most sensitive to environmental influences. No associations were reported between term LBW and trimester-specific exposures or entire pregnancy exposures to TTHM [9,10]. Investigators who were able to address variation in residential exposures observed a positive association between TTHM exposures and term LBW, decreased mean BW and increased risk of delivering a LBW infant despite low TTHM concentrations [29,30]. Others find a weak association of SGA with an exposure level of THM of 30 mg/L [31]. Some epidemiological studies reported a moderately increased risk of delivering a SGA infant among women exposed to high levels of TTHM, with relative risks ranging up to 1.5 [10-12,16].

An association between increased risk of intrauterine growth retardation and TTHM exposure was reported [10] and a dose-response trend was observed for exposure to chloroform [32]. Some authors did find a slightly elevated risk of intrauterine growth retardation during the second and third trimesters for TTHM [15] and others did not [33]. These studies differed in their exposure estimation because they mainly used the routinely measured DBPs levels in water and at times ingestion data. Lack of a consistent significant effect of the epidemiologic studies may be result of a study design, be a result of exposure misclassification or inadequate control for confounding variables, or a lack of power in studies sample, or actual lack of an effect of DBP on fetal growth.

A recent meta-analysis of epidemiological studies data on the association of TTHM concentration in water and fetal growth, without taking into account showering, bathing, and other exposure routes, concluded that there was little or no evidence for associations between TTHM concentration and fetal growth and that the uncertainties-relating particularly to exposure may have affected the results. The authors concluded that there is need a more accurate exposure assessment in the studies of the associations between TTHM and birth outcomes [34,35].

Only few studies have incorporated information on individual water use to estimate personal DBP exposure [16,19,36]. However, personal exposure analyzed as categorical variable did not show a stronger association than residential concentration with respect to fetal growth and fetal survival outcomes. These studies did not explore the effect of THMs as continuous variables on LBW or SGA risk. Recently, findings of a case-control study suggested that exposure to THMs at the highest levels can affect fetal growth but only in genetically susceptible newborns [37].

Our study offered advancement in individual internal dose assessment based on residential THM levels, detailed water use behaviors and exposure during pregnancy. Every subject's exposure indices were estimated as daily internal dose of the THM constituents (mg/d) and birth outcome effects were assessed by using indices categorical variable and also as a continuous variable. An additional strength of our study is that pregnant women were prospectively followed, and did not move during pregnancy. This allowed collection of self-reported data on potential confounding covariates. However, there is a possibility of residential confounding in our study, because we did not adjusted for e.g. residential air pollution exposure that might have effect on adverse birth outcomes [38]. An additional limitation of our study is because of lack information on maternal nutrition and infection diseases. This study exposure assessment also could be improved by more frequent measurement of DBPs at the every home tap and including other water usage activities and validation of data on DBP blood concentration measurement, but this is prohibitive expensive. Furthermore, lack of information regarding the validity of the internal dose assessment models that we used is one of the limitations of this study, but again validity studies are difficult to conduct and are expensive.

In this study, despite low concentrations of THM in drinking water, we found evidence of fetus growth restriction in mothers exposed to higher TTHM internal doses after controlling for family status, maternal education, chronic diseases, body mass index, blood pressure, smoking, alcohol consumption, previous preterm, infant gender, and year. However, the trihalomethanes are not the only by-products of chlorine disinfection or other contaminants, although in this region the levels of other by-products appeared to be very low, we cannot exclude the potential effects of this low-dose mixture, or any other related exposure.

The health effects of LBW and SGA are important issue for public health since these infants are at an increased risk of significant morbidity and mortality during the early stages of life.

## Conclusions

This study presented some epidemiological evidence for a dose-response relationship between THM internal dose exposure and LBW; a statistically significant association of THM with SGA was seen only for chloroform exposure. Our study used the questionnaire information to evaluate of pregnant women water usage habits and estimate integrated internal dose for THM exposure assessment. Our data showed that seeking to reduce exposure measurement errors in individual exposure determination, assigning exposure through dermal absorption, and inhalation should be considered combined with ingestion, since TTHM through ingestion composed less than 10% of integrated internal dose. This study finding suggest that internal dose in pregnancy vary substantially across individuals, depending on both water THM levels and water use habits and that internal dose may affect fetal growth. However, we do not feel this study provides strong support that any THM constituent is associated with fetal growth restriction. Futher research should focus on the use of integrated internal dose and individual susceptibility in the study of DBP effects on birth outcomes.

## List of abbreviations

BW: birth weight; CHBr_2_Cl: dibromochloromethane; CHBr_3_: bromoform; CHBrCl_2_: bromodichloromethane; CHCl_3_: chloroform; CI: confidence interval; DBP: disinfection by-products; LBW: low birth weight; OR: odds ratio; SGA: small for gestational age; TTHM: total trihalomethane; THM: trihalomethane

## Competing interests

The authors declare that they have no competing interests.

## Authors' contributions

RG and MJN conceived and designed the study, MKK oversaw the analytical work, SWK provided critical input into the manuscript and drafted the manuscript. JV performed statistical analysis, AD, GB and VK assisted with the writing manuscript. All authors read and approved the final version.
